# A Real-Life Based Evaluation of the Effectiveness of Antibacterial Fabrics in Treating Atopic Dermatitis

**DOI:** 10.1155/2018/7043438

**Published:** 2018-11-19

**Authors:** Dirk Höfer

**Affiliations:** Hohenstein Institute for Textile Innovation, Schlosssteige 1, 74357 Bönnigheim, Germany

## Abstract

**Background:**

Antibacterial clothes are classified as a complementary treatment in line with antisepsis, although meta-studies are unable to find significant improvements of eczemas.

**Methods:**

The antibacterial effectiveness of conventional AD clothes was compared across each other by (i) standard suspension tests for the appraisal of antibacterial products and (ii) a real-life setup of affected AD skin using* S. aureus* colonised artificial skin, to assess if functional clothes are effective under practical wear conditions. Additionally, the interaction of the fibre types with a moisturising cream was evaluated during a real wearing situation and after domestic laundry.

**Results:**

In the real-life setup simulating dry skin microenvironment, all samples failed to reduce* S. aureus. *Silver and zinc-fabrics showed a slight activity only under unrealistic moist conditions. When using standard suspension tests, samples differed considerably in their antibacterial effectiveness, where silver and zinc endowed fibres outperformed AEGIS endowed silk fabrics. Garments absorbed the cream dependent on the particular fibre types. Furthermore, domestic laundry was unable to completely remove the cream.

**Conclusion:**

Considerable differences in the antibacterial effectiveness of conventional AD clothes were revealed. Under practical (dry) wear conditions, garments were unable to modify skin colonization with* S. aureus*, although effectiveness can be triggered by wetting the garments. Remnants of moisturising cream remain on the fibres after laundry.

## 1. Introduction

Atopic dermatitis (AD) is a chronic, recurrent inflammatory skin disease with a significant social and economic burden. It has an estimated prevalence of up to 20 % in children and 2 % in adults [1, 2] and a considerable impact on the patient's quality of life, depending on the severity of the disease. AD is a multifactorial disease that is influenced by inheritance as well as by a variety of environmental factors. Its pathophysiology comprises immunological deregulations such as Th1/Th2 dysbalance and interleukin 31 as a key pruritic skin factor, leading to defects in skin barrier function like reduced water retention [3, 4]. The complex network of immunological deregulations may also explain the high rate of cutaneous colonization with* Staphylococcus aureus* (up to 90% in moderate to severe eczema) and* Staphylococcus epidermidis* in AD [5–7]. Only* S. aureus* is able to produce virulence factors such as super-antigens and therefore exacerbates the skin inflammation [8]. However, dysbiosis in skin microbiome of AD patients does not only implicate Staphylococcus species, but also implicate microbes such as* Cutibacterium spp.* and* Malassezia* [9].

Clothing plays a pivotal role as a provoking factor of AD. Therefore, patients carefully choose smooth clothing and avoid irritating fabrics and fibres like wool to prevent primary skin irritation [10, 11]. This insight led to the development of functional textiles. However, whereas some fabrics such as cotton and silk garments tend to reduce scratching by smooth fibre types, a subset of garments have been specifically designed, claiming to reduce the intensity of the eczemas by modulating the skin staphylococcal profile via an antibacterial activity [12, 13]. Some antibacterial clothes therefore use the deposition or incorporation of metallic silver or silver compounds in or onto synthetic fibres [14], whereas others use zinc or quaternary ammonium as antibacterial agents [15, 16]. Subsequently, a number of clinical trials (randomized controlled or observational) were conducted using antibacterial clothes for AD treatment, most of which claimed skin improvements in SCORAD, fewer symptoms, or reduced itching. However, a systematic meta-analysis study stated that these trials are of low quality of supporting evidence regarding the effectiveness in AD symptoms and severity: The test designs were unable to guarantee randomization of the test subjects, showed a small sample size, used different active agents in the respective clothes, or continued comedication with topical glucocorticoids, calcineurin inhibitors, or moisturising creams during the trial [17]. The meta-analysis study concluded that recommendation for the use of functional textiles in AD treatment is weak and that more studies with better methodology and longer follow-up are needed. Although, in the considered RCTs, AD patients mostly assessed functional clothes positively, it was unclear if this vote can be assigned to either the antibacterial effect, the use of smooth fibres, or, for example, a combination of both traits. In contrast to this, a Cochrane review on interventions to reduce* S. aureus* in the management of AD stated that topical antibiotics as well as antiseptics (including one trial using silver textiles) showed no significant improvements in eczema [18], a finding that was supported by a recent health technology assessment, which studied the effectiveness of oral and topical antibiotics [19].

Against this background, it is not surprising that medical guidelines pertaining to AD emphasize that antibacterial clothes could at least “potentially improve disease severity” in patients prone to noncommensal bacteria colonisation and skin barrier impairment [20]. Furthermore, health technology assessments merely summarized the effect of antibacterial clothes “to be considered as a complementary treatment.” In this situation, a situation full of contrasts, it might be helpful to study the effectiveness with a practical in vitro approach, for example, by using a* S. aureus*-affected skin model, in order to assess whether antibacterial effects of functional clothes on dystopic skin can be substantiated on a laboratory scale.

Besides clothing, the use of moisturisers is emphasized by healthcare professionals and the guidelines as a basic part in the treatment of AD. Moisturisation is suggested to enhance the healing of eczemas and to prolong the clinical improvement after discontinuation of anti-inflammatory therapy, thereby reducing the need for additional treatment, including topical corticosteroids [21–23]. Up to now, there is no data available if and how functional clothes and fibre types interfere with moisturisers. For instance, the presumed antibacterial effect of functional clothes might actually be disturbed by the simultaneous use of moisturising creams. On the other hand, cream remnants might cling to the clothes, mitigating the severity of eczemas.

In this respect, this research study conducted controlled in vitro tests: First, to compare the antibacterial effectiveness of five conventional functional clothes for AD treatment by quantitative international standard suspension tests. Second, as these normative approaches are not an accurate reflection of the environmental conditions under which AD garments are worn, a real-life setup of affected AD skin was used, based on artificial skin inoculated with* S. aureus (*i.e., a practical wear simulation of dysbiotic skin), to compare the clothes antibacterial activity in vitro. Finally, the interaction of the fibre types with a lipid-containing moisturising cream was evaluated during a real wearing situation and after domestic laundry.

## 2. Material and Methods

### 2.1. Fabric Characterisation

To objectively measure the potential antibacterial efficacy of antibacterial fabrics against a* S. aureus* strain, 5 commercial clothes were enrolled that have documented clinical benefits in treating atopic dermatitis in published reports: Sample #1 (Lyo-Zinc): Benevit Zinc+ (Benevit Van Clewe, Dingden, Germany) which consists of 74% lyocell, 19% SmartCell sensitive fibre, and 7% spandex [16]. Sample #2 (Silk-Aegis): Microair Dermasilk, a pure form of silk consisting exclusively of fibroin and containing a finish of AEGIS AEM 5772/5, an insoluble colorless, odorless ammonium as antibacterial agent [5, 13]. Sample #3 (PA-Silver): Padycare (Texamed, Ismaning, Germany), a micromesh material of 82% polyamide, 18% lycra with woven silver filaments with a silver content of 20% in total (130 g/m^2^) [12]. Sample #4 (Smart-Zinc): Smartcel sensitive consisting of 70% Supima cotton, 18% lyocell, and 12% elasthane [24]. Sample #5 (Modal-Silver): Binamed made of 79% modal, 14% silver yarn, and 7% lycra (210 g/m^2^).

Published reports were analysed comprising randomised controlled trials (RCTs) and observational and case-studies (with a cohort or case-control design) that compared the effects of patients with a clinical diagnosis of atopic dermatitis, irrespective of the age of the patients. Data was extracted exclusively with respect to the preclinical characterisation of the devices, especially, if standardised tests have been run to evaluate the antibacterial effectiveness.

### 2.2. Determination of Antibacterial Activity via Standards

The antibacterial activities of all textile samples were evaluated with the suspension test according to the standard ISO 20743:2007 “Textiles-determination of antibacterial activity of antibacterial finished products.” All tests were run in triplicate. The determination of the antibacterial activity was performed by the absorption method, in which a test bacterial suspension is inoculated directly onto samples. In brief, textile swatches were inoculated with a starting suspension of 10^7^ of* Staphylococcus aureus* (American Type Culture Collection 6538) obtained from the Deutsche Sammlung von Mikroorganismen und Zellkulturen (DSMZ, Braunschweig, Germany; since the resident skin microflora does not include gram-negative germs, we did not investigate the effect of the samples towards* Klebsiella pneumoniae, *another test germ of the standard). After 6 h, 18h, and 24 h of incubation at 37°C, the colony plate count method was used for the enumeration of bacteria colony forming units (CFUs; detection level < 20 cfu/sample). The specific antibacterial activity A was determined by inoculating a negative control PES fabric but without the antibacterial activity. The efficiency of the activity was then calculated by the following equation:

(1) $$\begin{array}{c} A=\left(\;\log_{10}C_{t}-\log_{10}C_{0h}\;\right)-\left(\;\log_{10}T_{t}-\log_{10}T_{0h}\;\right) \end{array}$$

where C = control material, T = sample, and t = time point.

The antibacterial effectiveness of the Silk-Aegis and Smart-Zinc samples was further evaluated with the dynamic shake flask test ASTM E2149-01 as recommended by published data [25]. Therefore, 1 gram of the textile was cut in small pieces and the whole amount was inserted in sterile flasks containing 50 ml of shake-flask buffer. Each flask was inoculated with a final concentration of 1-6 x 10^7^ CFU/ml of* S. aureus* and 1 ml of suspension was taken and used to assess the initial load of microorganism (defined as T0 load and expressed as CFU/ml). Flasks were then incubated under gentle shaking aerobic conditions for up to 24 hours at 37°C. At the end of incubation (6h, 18 h, or 24 h), 1 ml of the* S. aureus* was plated onto agar plates as described above. The antimicrobial activity of the Silk-Aegis and Smart-Zinc samples was evaluated according to the formula below [26, 27], where B represents the number of bacteria for blank (PES negative) and A is the number of bacteria for the samples after contact time:

(2) average  microbial  growth  reduction,log=log⁡B−log⁡A.

where A = cfu in the tested sample after x hour, B = cfu estimated in the starting suspension before the addition of the sample (0 h).

The general assessment criteria follow a definition by the ISO 20743 in that a growth reduction efficacy of <0.5 corresponds to no antibacterial activity, whereas ≥0.5 to <1 corresponds to slight, ≥1 to <3 to significant, and a growth reduction of ≥3 indicates a strong antibacterial activity, respectively. Standard ASTM: E2149 – 01 does not quote any criteria to define the level of antibacterial properties.

### 2.3. Real-Life Setup of Affected AD Skin

To simulate* S. aureus* affected human AD skin and to evaluate bacterial reduction during garment contact, a real-life setup of fabrics worn on inoculated artificial AD skin was developed. The test was designed to reflect normal conditions of use in terms of humidity, temperature, and contact frequency. In principle the setup followed the disc carrier test published by Ebert et al. [28]. As a replacement of metal disks, the standardized artificial skin VITRO-SKIN® N-19 (IMS Inc) was used. Pieces of VITRO-SKIN® N-19 were cut into squares of 3 cm^2^ and placed in chambers with 15% glycerine for 18 h in order to achieve humidity. 50 *μ*l of 1 x 10^8^ CFU/ml of a starting suspension of* S. aureus* in tryptone-NaCl were inoculated at 0 h on the artificial skin with a sterile glass spatula to gently spread the suspension, which was then dried for a short time and then covered with either antibacterial samples or nonantibacterial control textile swatches. The tests were run in triplicate either in dry (50% relative humidity at 30°C) or wet conditions. For the latter, all samples were prewetted prior to the contact with the skin for 1 min with PBS Phosphate-Buffered Saline (containing 137mM NaCl, 2.7mM KCl, and 10mM phosphate, pH 7.4) to allow the samples to be completely wetted with the solution and to become saturated with it. It was ensured that all textile samples had a close fit to the skin by placing a glass slide on top of each sample so that they were permanently covering the artificial skin. In addition, the relative water content of all samples was measured under standard climate prior and after wetting the samples in PBS for 1 min.

The antibacterial activity of the textile swatches against* S. aureus* was analysed quantitatively on the artificial skin over a contact period of up to 18 h and assessed against the internal positive control Biatin AG® (Coloplast GmbH, Hamburg, Germany), a conventional foam dressing containing homogeneously distributed silver which continuously releases silver. A PES material without antibacterial activity served as negative control. If a significant germ reduction appeared within this wearing/exposure period, the textile was regarded as effective with respect to effects towards the human skin flora. Bacterial solutions were collected from the artificial skin immediately after the exposure times (1 h, 4h, and 18 h). For that, the remaining flora was collected by elution of the skin with 2 mL of sterile NaCl with 0.2% Tween 80. 100 *μ*L was plated on agar medium. Plates were incubated at 36°C under aerobic conditions and inspected after 2 days. The number of colony forming units (CFUs)/200*μ*l was determined using a colony counter (IUL Instruments GmbH, Koenigswinter, Germany). The efficiency of the activity was then calculated analogous to the ISO 20743.

### 2.4. Emollient Application and Wear

The reference moisturising cream for the emollient application used in this study was Linola Fett (Dr. Wolff, Bielefeld, Germany), a W/O emulsion with a lipid content of 60%. Linola Fett contains unsaturated fatty acids (including linoleic acid) which are needed for the barrier function in healthy skin and is prescribed for patients suffering from AD. According to the instructions of the manufacturer, the upper arms of a test person, which was checked for absence of any* S. aureus* colonization, were creamed after taking a shower with a recommended quantity of cream (2 cm per 130 cm^2^ of skin). Then 8 cm x 8 cm textile swatches were placed over the skin area fixed with a loose sleeve. After 4 h, the specimens were removed and further processed to either gravimetric lipid determination, domestic laundry, or the experimental model of inoculated AD skin.

### 2.5. Domestic Laundry and Gravimetric Lipid Determination

In order to exclude cross contamination with lipids, standardised small-scale laundry laboratory tests were conducted. Laundry was carried out in a Linitest according to ISO-105-C06-A1S with some modifications for temperature and detergent concentration, according to the fabric manufacturer's specifications for care instructions of each sample, that is, at either 30°C or 60°C, for 30 min. As detergent solution, the standard IEC detergent was used (4.0 g/l; 150 ml per sample). Disinfectants, chlorine-based products as well as fabric softeners, and ballast load were omitted. After laundry, all specimens were rinsed twofold with H_2_O for 2 min and finally air-dried. For the gravimetric lipid determination, all specimens were subjected into a round bottom flask after the wearing period. Following this, 250 ml of hexane was added and the bottom flask attached to a Soxhlet extraction apparatus. After 3 h extraction, the amount of lipid was recovered and its percentage in the original sample was calculated gravimetrically:

(3) Mass  of  lipid=weight  of  the  flask+boiling  chips+extracted  oil−weight  of  the  flask+  boiling  chips.The  lipid  content  %=mass  of  lipid  extracted gsample  weight g×100.

## 3. Results

For the five selected antibacterial clothes, a total of ten clinical studies concerning treatment of AD were found [5, 12, 13, 15, 16, 29–33]. These reports investigated as main endpoint the colonisation with* S. aureus* by swabbing the skin at affected skin sites of AD patients before and after the application of the clothes. All studies, except one [16], excluded a basic verification of the devices according to international standards to prove their antibacterial effectiveness in vitro. Thus, a lack of data was found concerning the comparability or equivalence of the products in terms of their antibacterial effectiveness as well as the clinical evaluation of the products on the basis of the current state of the art. However, all studies claimed mitigating effects of clothes in AD due to the respective antibacterial agents. Four studies continued comedication with topical glucocorticoids, calcineurin inhibitors, or moisturising creams during the trials.

In order to evaluate the antimicrobial effectiveness of each sample and to compare the results with the other conventional devices, the antibacterial activity of all samples was evaluated using the international standard ISO 20743 in a time-trend comparison. In these suspension tests, the five samples considerably differed in their respective antibacterial activity. As expected, an internal silver-containing antibacterial PES material, used for validation over time, resulted in constant 6-log-step reduction of* S. aureus *over 24 h. According to the standard method ISO 20743, Lyo-Zinc and PA-Silver exhibited strong antibacterial activities of almost 6 log steps within the first 6 h and kept it at constant levels at least for 24 h. In contrast to this, Smart-Zinc started with a lesser antibacterial activity of 4 log steps at 6 h that was kept at constant level within the next 18 h. The biocidal activity of Modal-Zinc corresponded with Smart-Zinc of showing a significant activity at 6 h and keeping it constant over 18 h and 24 h (Table 1, left side). With an approximately 1.6-log-step reduction, the non-silver or zinc containing Silk-Aegis sample displayed a much weaker antibacterial activity following the ISO 20743 protocol. Interestingly, this sample failed to exhibit a microbial reduction in the shake flask test ASTM E 2149-01 over the entire period, although the shake flask test is recommended for the respective biocide AEGIS. In contrast to that, the Smart-Zinc sample showed a strong activity, which was even slightly higher in the ASTM setup compared to the ISO standard (Table 1, right side).

As quantitative suspension tests are not an accurate reflection of the environmental conditions under which AD garments are worn on a patient's skin, a real-life setup of affected AD skin was used, based on artificial skin inoculated with* S. aureus,* to assess the effectiveness of antibacterial textiles in vitro towards a dysbiotic human skin flora. In contrast to the ISO and ASTM methods, in this setup none of the samples displayed a significant antibacterial effect over a time period of 4 h and 18 h when the clothes were worn at 50 % relative humidity at dry skin environment and in close contact on the artificial skin (see Table 2). However, when the samples were wetted to their individual water absorption capacity and left on the skin for 18 h, the zinc-containing fabrics (Lyo-Zinc and Smart-Zinc) displayed considerable stronger antibacterial activities than the silver-containing samples made of modal and polyamide. Although the wearing of wetted AD clothes is unrealistic in real life for AD patients, we explored in this setup the onset of the antibacterial action towards the* S. aureus* colonised skin. The positive control of wetted Biatain Ag reduced the inoculum after 1 h to about 1.42 log steps and after 4 h to about 4 log steps. Besides Biatain Ag, none of the other samples exerted a significant activity over 1 h under wet wearing conditions. The wetted PA-Silver sample at least showed a 2-log-step reduction after 4 h of application.

Fabrics were further evaluated for their respective lipid absorption capacity after a 4 h wear period with the reference moisturising cream. The Silk-Aegis fabric absorbed the cream slightly to more than 30% of its own weight (310 mgr of cream/gr fabric), whereas the Lyo-Zinc sample, PA-Silver, Smart-Zinc, and Modal-Silver samples showed a rather fibre independent uptake of the moisturising cream. After laundering of the fabrics, there was still a considerable lipid load present on all samples (see Table 3). Washing at 30°C did not remove the moisturising cream and left a considerable quantity on the samples, whereas washing at 60°C reduced the lipid load to about 5%.

## 4. Discussion

To the best of the author's knowledge, no in vitro study has been done across different garments to compare the effectiveness of conventional antibacterial clothes in AD treatment. All RCTs and observational studies used for the analysis therein [5, 12, 13, 15, 16, 29–33] excluded a basic verification of the respective fabrics according to international standards to determine the antibacterial activity of the textile product [25]. Products thus were unfortunately not comparable to each other across the studies. However, all trials claimed mitigating skin effects to the respective antibacterial agents, like SCORAD improvement or reduced* S. aureus* colonisation at affected skin sites, although no sample was subjected to a comparative in vitro approach to prove the claimed biotic action.

This research first compared the effectiveness of functional AD clothes by quantitative suspension standard tests to prove the antibacterial activity of the conventional fabrics quantitatively. The fabrics differed considerably in their antibacterial properties. The silk-based sample endowed with the biocide AEGIS showed only a weak activity according to the standard ISO 20743 and even failed when using the recommended dynamic shake flask test ASTM E2149-01 [25]. These results are in close correlation with previous in vivo findings by Ricci et al. [15], who were unable to demonstrate that silk fabrics coated with AEGIS AEM 5572/5 have an antibacterial activity by counting bacteria of the antecubital area of AD patients. However, the authors (and other studies using this kind of silk material) referred to an in vitro activity of the silk fabric which goes back to a single industry-led research in 1978 [34]. In case of standard ISO 20743, the antibacterial effectiveness of the silver-coated polyamide and the zinc-containing lyocell fibre performed best among the samples, whereas the silver-containing modal fibre and the cotton/lyocell samples displayed somewhat weaker antibacterial activities over 24 hours. Thus, to a certain extent, the zinc-containing sample confirmed its antibacterial activity in vitro, as shown for this material by Wiegand et al. [16], albeit the present study found markedly lower reduction values.

A first reason for the different performances by the samples is explained by the technical setup of the normative ISO and ASTM approaches. It is generally accepted that standard tests favour outcomes of antibacterial activities by creating favourable moist and warm conditions. That is why Kramer et al. in 2006 already encouraged further development of international standards for the in vitro testing and preclinical evaluation of efficacy and tolerance of hygienic AD clothes [26]. In their view, the mere product claim does not indicate a true effect and should be demonstrated independently for each device. Following the idea of Kramer, this research therefore simulated a real-life wear setup in vitro, using artificial skin colonised with* S. aureus,* to evaluate and compare the advantages of the antibacterial properties for the intended use of correcting a dysbiotic AD skin. The real-life setup proved to be a useful and easy-to-apply method to evaluate the antibacterial effectiveness of antimicrobial clothes in direct contact to skin. The test reflected normal conditions of use in terms of humidity (50% rH), temperature (30°C), and contact frequency. Furthermore, the test method allowed a more realistic assessment simulating conditions on the AD skin, that is, an evaluation of the staphylococcal profile under different environmental wear conditions. It also imposed more stringent requirements on antibacterial effectiveness over time than a quantitative standard suspension test, since contact between the garment and the test organism suspension to which the fabric is exposed is less intense on the artificial skin than during a suspension test, where samples are soaked with the inoculum. Furthermore, the normative approaches use test conditions with significant amount of liquid to allow diffusion of the incorporated agents to test organisms at planktonic and dynamic conditions and also provide optimum temperature conditions for the microbial growth.

In contrast to the ISO and ASTM methods, in the real-life setup none of the samples displayed a significant antibacterial activity after a wear period of 4 h and 18 h when the clothes were worn at a realistic skin microenvironment, that is, relative humidity of 50% and in close contact with the inoculated skin. In terms of the dermatological point of view, wearing of wetted AD clothes is unrealistic in real life that would hardly be tolerated by AD patients. Despite this fact, when the samples nevertheless were wetted to their individual water absorption capacity and left on the skin for 18 h, the zinc-containing fabrics (Lyo-Zinc and Smart Zinc) displayed stronger antibacterial activities than the silver-containing samples made of modal and polyamide. This observation supports the fact that moisture is needed to release a sufficient amount of contact biocide from antibacterial fibres. That is why the standard ISO 20743 is assessed to often overestimate antibacterial properties and to seem unsuitable to evaluate devices for practical applications. The poor antibacterial effectiveness of the samples in the real-life setup subjected to the dry skin microenvironment is corroborated by a previous placebo-controlled side-by-side study [35], in which 60 healthy human volunteers wore either of the functional clothes (silver-finished or silver-doped) of similar structure and with strong antibacterial activity according to standard methods for six weeks. In this field trial, the antibacterial halves did not disturb the skin microbiome in either germ number or composition and thus displayed no adverse effects on the ecological balance of the healthy human skin microflora. In the real-life setup simulating an affected skin, even under wet wearing conditions none of the samples exerted a significant antibacterial activity after 1 h; the PA-Silver fabric was merely effective after 4 h.

To the best of the author's knowledge, none of the RCTs and observational studies on functional clothes discussed the mechanisms of the presumed effect of silver or other biocides towards a selective eradication of* S. aureus* on dysbiotic skin. Daeschlein et al. carried out a trial with AD patients that wore silver-containing antibacterial fabrics for one week, to see whether silver impregnation prevents the bacterial growth within the textile [36]. Unexpectedly, this group found high residual contaminations of* S. aureus* despite silver exposure present on the fabrics after a wear period of at least 2 days. Their finding supports the view that a selective eradication of* S. aureus* by antibacterial clothes is highly unlikely.

Against the background that many in vivo RCT trials at affected AD skin sites of patients observed a bacterial shift to a reduced* S. aureus* colonization of functional clothes and on the basis of the work presented here, it cannot be ruled out that the bacterial shift may result by a nonantibacterial mechanism of action, that is, secondary effects. For example, Nakatsuji et al. recently showed that the reintroduction of human skin commensal bacteria like* S. epidermidis* to human subjects with AD protects effectively against a* S. aureus* colonisation, most probably by secretion of antimicrobial peptides AMPs [37]. This finding demonstrates a possible self-regulating role of the skins microbiome and concurrently opens up a new therapeutic field in which probiotics like Extracellular serine protease (Esp)-secreting* S. epidermidis* could improve AD symptoms. Furthermore, modulation of the skin microbiome by antimicrobial clothes might alternatively result through the enhancement of the activity of topic pharmaceuticals, that is, by prolonging the pharmaceutical effect [27] or simply by reducing scratching effect and itching, as known from woollen clothes [11], rather than by an intrinsic antibacterial activity. This view is supported by the observation within this study that the silk sample displayed no antibacterial effect at all, but absorbed a significantly greater quantity of the moisturising cream. The drug prolongation effect might be an additional reason for the observed SCORAD improvements observed in many trials conducted with silk fabrics [5]. However, a RCT with over 300 affected children run by Thomas et al. concluded that the addition of silk garments to standard AD care is unlikely to improve AD severity, or even to be cost-effective compared with the standard care alone, for children with moderate or severe AD [38].

Finally, the fibre type might as well have an influence on the dysbiotic skin. Callewaert et al. found that, depending on the fibre, a selective bacterial enrichment takes place, resulting in another textile microbiome as compared to the autochthonous skin microbiome [39]. Accordingly, the researchers observed no bacterial enrichment on viscose, but an enrichment of* Staphylococcus epidermidis spp*. and* Micrococci* on both cotton and polyester textiles. Other authors reviewed that different textile components are associated with different effects on the skin [10, 11]. Silver-coated cotton, for example, seemed to be more effective in decreasing lesion severity, while silk fabrics appeared to be more likely to alleviate pruritus and symptoms. Silk clothes may affect overall disease status by improving comfort and reducing itch sensation or by cooling the AD skin. It is noteworthy that all man-made fibres of the selected functional clothes in this study were optimized in terms of skin friction: They have round cross sections and smooth fibre surfaces with excellent comfort and thermoregulatory properties, which, by their structures, diminish the physical movements and by this way disrupt the itch-and-scratch cycles. The same holds true for the natural silk filament. Therefore, more studies are needed to better understand the interactions of functional fabrics with the skin microbiome of healthy and affected subjects and to better understand why these clothes mitigate disease severity, symptoms, and quality of life [17].

Emollients are the mainstay of maintenance of AD therapy [40]. Therefore, it has to be taken into account that in the daily routine there is a steady contact of functional clothes with topical medications. In this study, the AD clothes also differed in their absorption capacity to the moisturising cream, most probably according to the physicochemical nature of the respective fibre types. Thus, the data affirmed the importance of the interactions of fabrics and emollients in the management of AD. For moisturising creams contain also other excipients, such as emulsifiers, pH-adjusters, chelators, and preservatives, these excipients—but also moisturisers like urea, or pharmaceuticals like glucocorticoids or calcineurin inhibitors—might as well interfere with the microbiome. It was also found that laundry according to care instructions of the manufacturers was unable to eliminate the lipids. In daily practice this might lead to an accumulation of lipids over laundry cycles that at least contributes to the skin care. Only one study so far, of Daeschlein et al., examined the effect of washing of AD clothes. The group investigated whether silver impregnation of fibres at least prevents bacterial growth within the textile [36]. Unexpectedly, they found high residual contaminations despite silver exposure. The authors concluded that the risk of a recontamination source of* S. aureus* could be eliminated by machine-based washing at 60°C using conventional washing powder. However, although 60°C safely eliminates germs in contaminated clothes, it could be ineffective in removing the remnants of a lipid-based moisturising cream.

## 5. Conclusion

In this research, the antibacterial effectiveness of five conventional functional clothes for AD treatment was compared. In a real-life setup simulating practical wear conditions of a dry skin microenvironment, AD clothes were unable to modify the skin colonisation with* S. aureus*. Although effectiveness can be triggered by wetting the garments, this is however contraindicated for AD patients in their everyday life. When using normative suspension tests, samples showed some antibacterial activities. Garments absorbed moisturising cream dependent on the respective fibre type and remnants of cream still remained on the fibres after laundry. More studies are needed to better understand the interactions of functional fabrics with the skin microbiome of healthy and affected subjects.

## Figures and Tables

**Table 1:** Time-trend comparison of antibacterial effectiveness of conventional AD clothes against *S. aureus* according to the standards ISO 20743 and ASTM E 2149-01. Results depicted as log 10 reduction factors, means of triplicates with SDs. The ISO setup revealed a strong antibacterial effect for the silver-polyamide and the zinc-containing lyocell fabrics. The weakest activity was obtained with the Silk-Aegis sample. Right side: Evaluation of the antibacterial activity of the Silk-Aegis sample via ASTM test. Note failure of activity for the silk garment.

	ISO EN	20743		ASTM E	2149-01	
	Microbial [log cfu]	Activity	A	Microbial [log cfu/ml]	growth	reduction
	6 h	18 h	24 h	6 h	18 h	24 h
PES negative	0 ± 0.1	0 ± 0.1	0 ± 0.1	0 ± 0.1	0 ± 0.1	0 ± 0.1
PES positive	5.89 ± 0.1	6.29 ± 0.2	6.37 ± 0.2			
Lyo-Zinc	5.89 ± 0.1	6.29 ± 0.2	6.37 ± 0.2			
Silk-Aegis	2.23 ± 0.1	1.64 ± 0.1	1.54 ± 0.1	-0.03 ± 0.1	0.36 ± 0.1	-0.23 ± 0.1
PA-Silver	5.89 ± 0.1	6.29 ± 0.2	6.37 ± 0.2			
Smart-Zinc	3.96 ± 0.2	4.22 ± 0.2	3.54 ± 0.1	0.92 ± 0.2	6.53 ± 0.1	6.02 ± 0.1
Modal-Silver	4.12 ± 0.1	3.73 ± 0.1	3.91 ± 0.2			

**Table 2:** Comparison of antibacterial activity of conventional AD fabrics subjected to the *S. aureus* inoculated artificial skin. Results depicted as log 10 reduction factors, means of triplicates with SDs. The zinc-containing samples displayed considerable stronger antibacterial activities after 18 h followed by the silver-containing samples, but only when the samples were prewetted to their individual water absorption capacity. No short-term effect (1 h) of wetted samples on colonized skin was observed.

		Real-Life	Setup of	affected	AD skin	
	Water absorption	Microbial	Growth	Activity	[log cfu]	
	[g/swatch]	1 h wet	4h wet	4 h dry	18 h wet	18 h dry
Biatain Ag	5.70	1.42 ± 0.1	3.96 ± 0.1	0.85 ± 0.1	3.13 ± 0.1	2.65 ± 0.1
PES negative	0.41	0 ± 0.1	0 ± 0.1	0 ± 0.1	0 ± 0.1	0 ± 0.1
PES positive	0.46	0.15 ± 0.1	0.21 ± 0.2	0.50 ± 0.1	0.15 ± 0.2	0.17 ± 0.2
Lyo-Zinc	0.68	0.11 ± 0.1	0.62 ± 0.2	0.39 ± 0.2	2.99 ± 0.2	-0.67 ± 0.1
Silk-Aegis	0.14	-0.2 ± 0.1	-0.22 ± 0.1	-0.16 ± 0.1	-0.19 ± 0.1	-1.33 ± 0.2
PA-Silver	0.07	0.14 ± 0.1	2.09 ± 0.2	0.47 ± 0.2	1.75 ± 0.2	-0.58 ± 0.2
Smart-Zinc	0.64	0.11 ± 0.2	0.50 ± 0.2	0.31 ± 0.1	2.02 ± 0.2	-0.65 ± 0.1
Modal-Silver	1.23	0.25 ± 0.1	0.93 ± 0.1	0.43 ± 0.2	1.51 ± 0.2	-0.56 ± 0.1

**Table 3:** Lipid absorption values of five AD fabrics after wearing a moisturising cream for 4 h and after laundry. All values are means of triplicates and shown in mgr/gr of textile swatch.

	Lyo-Zinc	Silk-Aegis	PA-Silver	Smart-Zinc	Modal-Silver
After wear	171	310	196	173	125
After laundry	168	260	46	74	48

## Data Availability

The microbiological and antibacterial activity, the laundry, and gravimetric data used to support the findings in this study are included within the article. The microbiological and antibacterial activity, the laundry, and gravimetric data used to support the findings of this study are available from the corresponding author upon request.
